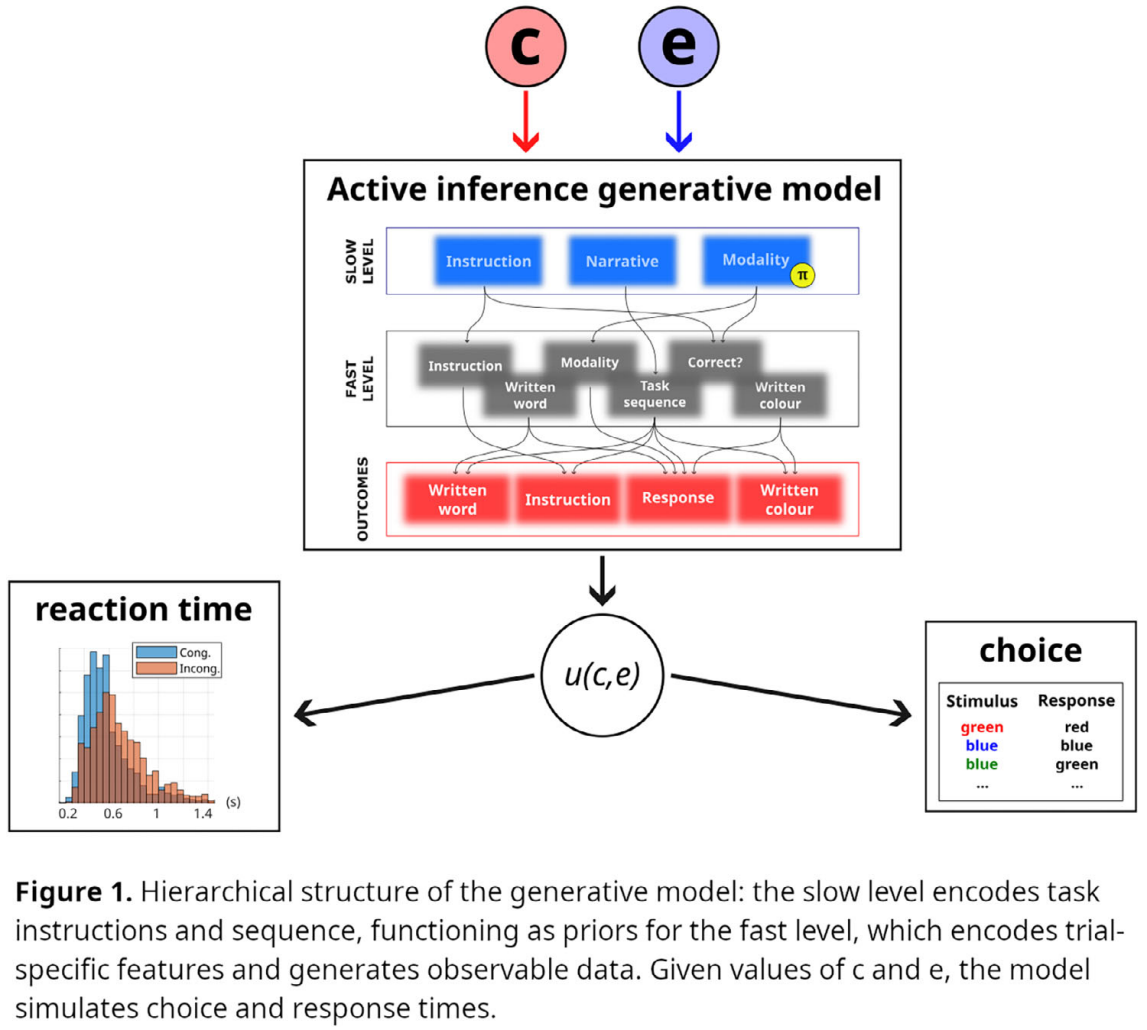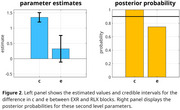# Voluntary modulation of mental effort: an Active Inference model

**DOI:** 10.1002/alz70855_103687

**Published:** 2025-12-24

**Authors:** Riccardo Maramotti, Thomas Parr, Manuela Tondelli, Daniela Ballotta, Giovanna Zamboni, Giuseppe Pagnoni

**Affiliations:** ^1^ Università di Modena e Reggio Emilia, Modena, Italy; ^2^ University of Oxford, Oxford, United Kingdom

## Abstract

**Background:**

Mental effort is a core component of cognitive control, encompassing two key dimensions: exogenous effort (automatic recruitment of cognitive resources; Kahneman, 1973) and endogenous effort (intentional modulation of engagement; Muraven & Baumeister, 2000). This study explores the role of voluntary mental effort in cognitive control using a computational model of the Stroop task. In the Stroop task, participants are presented with colored words (e.g., the word “blue” in red font) and must report the font color while suppressing the automatic tendency to read the word, making the task cognitively demanding.

**Method:**

Twenty healthy volunteers (mean age 27.9 ± 5.7 years) were asked to perform the Stroop task on a computer, alternatively with maximum exertion (EXR) or in a relaxed‐as‐possible fashion (RLX; Khachouf et al., 2017). The participants’ behavior was modeled with a two‐layer generative model grounded in the active inference framework (Parr et al., 2022). This approach enables the estimation of two key parameters: e, representing the strength of individual bias toward reading words (versus reporting colors), and c, reflecting individual motivation during task performance (Figure 1).

**Result:**

The EXR condition was associated with a significant increase in the motivation parameter but not in the bias parameter (Figure 2). These findings suggest that voluntary engagement of mental effort primarily enhances inner motivation rather than directly altering habitual response tendencies. Since the only difference between EXR and RLX conditions was the instruction to modulate effort, the observed behavioral changes can be attributed to variations in endogenous effort.

**Conclusion:**

This study has important clinical implications, as deficits in effort regulation are common in neurodegenerative disorders such as Alzheimer's and Parkinson's disease. Furthermore, this computational approach offers a novel framework for inferring internal cognitive and motivational states from behavioral performance, with potential applications in the diagnosis and treatment of psychiatric and neurological conditions.